# Overview of the Ultrasound Classification Systems in the Field of Thyroid Cytology

**DOI:** 10.3390/cancers13133133

**Published:** 2021-06-23

**Authors:** Esther Diana Rossi, Liron Pantanowitz, Marco Raffaelli, Guido Fadda

**Affiliations:** 1 Division of Anatomic Pathology and Histology, Fondazione Policlinico Universitario Agpstino Gemelli, 00168 Rome, Italy; guido.fadda@unime.it; 2 Department of Pathology & Clinical Labs, University of Michigan, Ann Arbor, MI 48103, USA; lironp@med.umich.edu; 3 Division of Endocrine-Surgery, Fondazione Policlinico Universitario Agpstino Gemelli, 00168 Rome, Italy; marco.raffaelli@policlinicogemelli.it; 4 D.A.I. Diagnostic Department of Anatomic Pathology, University of Messina, 98100 Messina, Italy

**Keywords:** thyroid, classification system, follicular neoplasm, ultrasound classification system, TIRAD

## Abstract

**Simple Summary:**

Ultrasound (US) is the preferred imaging modality for thyroid nodule evaluation. Accurate US assessment of thyroid lesions can help decrease unwarranted FNA procedures of benign nodules. Several thyroid nodule risk classification systems that focus on US features have been published. Some of them highlight simple US patterns, while others rely on the presence of multiple US features to categorize thyroid nodules. The current review offers an evaluation of different US system, combining them with the use of fine needle aspiration and the cytological classification systems.

**Abstract:**

The increasing application of ultrasound (US) in recent years has led to a greater number of thyroid nodule diagnoses. Consequently, the number of fine needle aspirations performed to evaluate these lesions has increased. Although the majority of thyroid nodules are benign, identifying methods to define specific lesions and tailor risk of malignancy has become vital. Some of the tools employed to stratify thyroid nodule risk include clinical factors, thyroid US findings, and reporting systems for thyroid cytopathology. Establishing high concordance between US features and cytologic diagnoses might help reduce healthcare costs by diminishing unnecessary thyroid procedures and treatment. This review aims to review radiology US classification systems that influence the practice of thyroid cytology.

## 1. Introduction

Thyroid nodules are common in adults. In recent years, the incidence rate of thyroid cancer has increased, as has the rate of thyroidectomy [1,2]. However, the overall mortality for thyroid malignancy during this time period showed no significant changes. The increase in diagnosing thyroid lesions is partly attributed to improvements in imaging technology and increased use of imaging, which leads to higher rates of thyroid nodule detection [3,4,5]. As a result, finer needle aspiration (FNA) biopsies and, accordingly, a higher incidence of subclinical thyroid cancer has risen. FNA is the first and perhaps most important minimally invasive diagnostic tool employed in the evaluation of thyroid nodules [6,7,8,9,10,11]. Around 70% of thyroid nodules are benign, with only 5–10% reported to be malignant [1,2]. The remaining 20–25% of thyroid lesions comprise grey zone indeterminate proliferations that include either benign or malignant lesions, for which morphological discrimination alone is not always possible. These aspects raised concerns over the costs and morbidity linked with the management of patients with thyroid nodules. On the whole, it often leads to unnecessary surgical resections and drives up healthcare cost. It stands to reason that a more refined and accurate approach to the management of thyroid lesions needs to start from an accurate initial workup including US evaluation, avoiding the over-diagnosis of low-risk lesions [2,3,4,5,6].

According to the American Thyroid Association (ATA), ultrasound (US) is the main and preferred imaging modality for thyroid nodule evaluation [7]. Accurate US assessment of thyroid lesions can help decrease unwarranted FNA procedures of benign nodules. Several thyroid nodule risk classification systems that focus on US features have already been published. Some of them highlight only simple US patterns, while others rely on the presence of multiple US features to categorize thyroid nodules. In 2009, Horvath et al. proposed a Thyroid Imaging, Reporting and Data System (TIRADS) [12] (Table 1) accepted and then proposed by the American College of Radiology (ACR) and based upon the distribution of US features in five categories (composition, echogenicity, shape, margin, and echogenic foci) [13,14]. The TIRADS reporting system has notably been modeled after the 2009 Breast Imaging Reporting and Data System (BIRADS) [15].

In 2015, Grant et al. published a thyroid ultrasound reporting lexicon in which all thyroid nodules were classified on the basis of TIRADS categories which, in turn, not only defined their risk of malignancy but offered evidence-based recommendations to manage thyroid nodules based on their size and sonographic features [5]. After the first Korean version of the TIRADS system by Kwak et al. [14], Shin et al. (2016) subsequently proposed a revised Korean Society of Thyroid Radiology (KSThR) consensus statement with recommendations in which specific sonographic features were used to stratify the risk of thyroid nodules into four categories [8]. According to the published literature, the Korean-TIRADS has been successfully used for US evaluation of thyroid nodules in order to stratify the need for these nodules to undergo FNA (Table 1).

The 2015 ATA guideline includes a detailed description of sonographic features, categorizing thyroid nodules that utilize one of the described patterns [7]. The most suspicious US features include margins, microcalcifications, “taller-than-wide” shape”, rim calcifications, and evidence of extrathyroidal extension. Specifically, the ATA defined and identified five categories: (1) Benign (ROM < 1%); (2) very low suspicion (ROM < 3% in lesions ≥ 20 mm); (3) low suspicion (ROM 5–10% in lesions ≥ 15 mm); (4) Intermediate suspicion (ROM 10–20% in lesions ≥10 mm); and (5) high suspicion (ROM 70–90% in lesions ≥ 10 mm).

Furthermore, the European Thyroid Association (ETA) TIRADS, which includes five categories, was published in 2017 by Russ et al., with the main purpose of identifying thyroid malignancies while maintaining both high negative predictive value and sensitivity [16]. Since then, several similar systems have been promoted including the recommendations from the American Association of Clinical Endocrinology (AACE), American College of Endocrinology (ACE), Associazione Medici Endocrinologi (AME), as well as comprehensive cancer network guidelines [17,18,19].

The current article reviews these different US classification systems and the influence they have on the practice of thyroid cytology.

## 2. Overview of ACR-TIRADS

In an attempt to stratify the risk of thyroid cancer utilizing US features, the TIRADS imaging risk stratification system was proposed by Horvath et al. from Chile in 2009 and further modified by Kwak et al. from Seoul in 2011 [12,14]. TIRADS is now accepted by the ACR and has been described in a paper published by the ACR TIRADS Committee [5].

**Figure 1:**
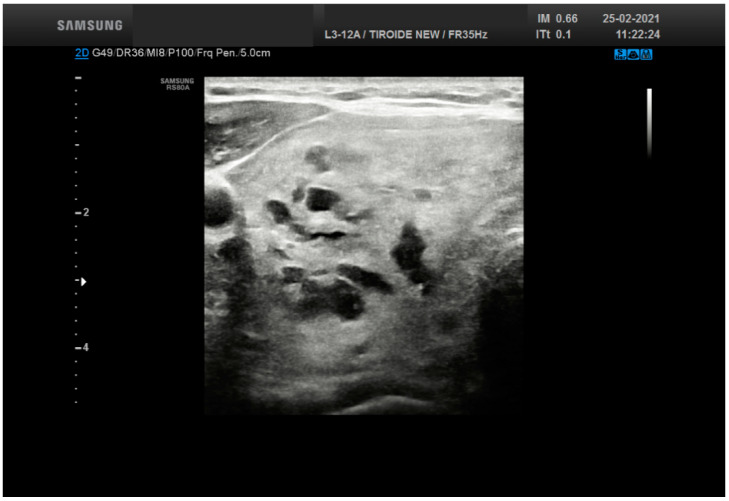
Ultrasound features from a thyroid nodule (50 mm size) resulting into a score 2 (solid and cystic plus hyperechoic-isoechoic) belonging to TR 2. The lesion was diagnosed as adenomatous goiter.

**Figure 2:**
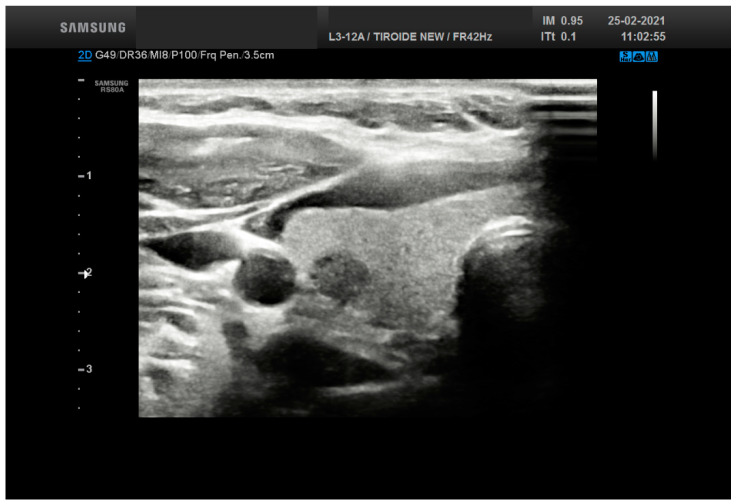
Ultrasound features from a thyroid nodule (15 mm size) resulting into a score 5 (solid, hypechoic with a calcification) belonging to TR 4. The lesion was diagnosed as Follicular nodule.

**Figure 3:**
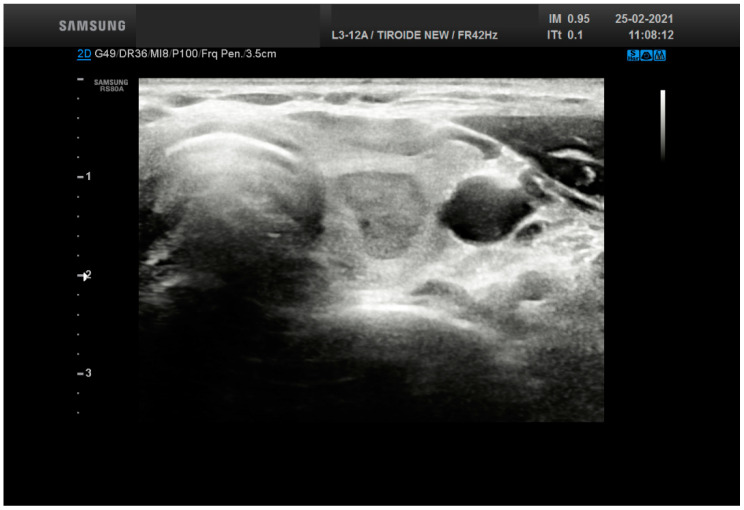
Ultrasound features from a thyroid nodule (15 mm size) resulting into a score 7 belonging to TR 5 (solid, very hypoechoic, lobulated). The lesion was diagnosed as Follicular nodule.

The ACR-TIRADS is designed to reduce the number of unnecessary FNA procedures performed for benign thyroid nodules with an objective to increase the diagnostic efficacy of evaluating thyroid nodules. The idea behind this system is to codify all thyroid lesions into diagnostic US categories. Specifically, five different US characteristics of a thyroid nodule are evaluated, including: (a) composition, (b) echogenicity, (c) shape, (d) margin, and (e) echogenic foci (Table 2). Points are assigned to each of these US features. For composition, the values are as follows: cystic or spongiform = 0, mixed solid-cystic = 1, and solid = 2. For echogenicity, they are anechoic = 0, isoechoic or hyperechoic = 1, hypoechoic = 2, and very hypoechoic = 3. For shape, wider-than-tall = 0, whilst taller-than-wide = 3. Margins are classified as follows: smooth or ill-defined = 0, irregular or lobulated = 2, and extrathyroidal extension = 3. The echogenic foci are classified as: none or comet-tail = 0, macrocalcifications = 1, peripheral or rim calcifications = 2, and punctate = 3. Points are totaled by adding single selections from the five nodular characteristics and they are then used to classify thyroid nodules into TIRADS categories as follow: TR1 = Benign (requires no FNA), TR2 = not suspicious for malignancy (requires no FNA-Figure 1), TR3 = mildly suspicious (FNA if ≥2.5 cm and follow if ≥1.5 cm), TR4 = moderately suspicious (FNA if ≥1.5 cm and follow if ≥1.0 cm-Figure 2), and TR5 = highly suspicious (FNA if ≥1.0 cm and follow if ≥0.5 cm-Figure 3). Concerning TR4, there was a further subclassification including TR4a with one malignant sign and possibly benign; TR4b with two malignant signs and possible malignant; TR4c with three or four malignant signs and highly possible malignant. Furthermore, the TIRAD Committee underlined the risk of malignancy (ROM) for each category as follows: 2% or less for TR1 and TR2, 2.1–5% for TR3, 5.1–20% for TR4, and greater than 20% for TR5. As indicated, the categories along with thyroid nodule size help determine recommendations for FNA and follow-up management.

The novelty of ACR-TIRADS is the method of scoring both echogenic foci and calcifications, which are additive features given more weight than in other systems. Some authors have suggested modifying TIRADS [13,20,21,22,23,24]. Park et al. established a new system with 12 characteristics even though its application proved to be difficult, [13] and Kwak et al. proposed a more practical classification system including only five US features [14]. Several studies have evaluated the efficacy of ACR-TIRADS [20,21,22,23,24]. Among the others, Koseoglu et al. documented that in a series of 2847 patients who underwent FNA of their thyroid lesions ACR-TIRADS was able to classify 98.8% as benign nodules with only a minimal number of malignant lesions classified as TR2 and TR3 [20]. Ha et al. compared seven society guidelines, of which ACR-TIRADS resulted in the lowest rate (25.3%) of unnecessary thyroid FNA procedures [21].

## 3. TIRADS Challenges and Pitfalls

The implementation and adoption of any new classification system are likely to present some challenges [2,3,4,5,6]. For ACR-TIRADS, such issues were mostly due to education, workflow, and interpretation of this reporting system. An initial step in the global adoption of a unique classification such as TIRADS is the education and training of sonographers to recognize the relevant US features. In general, a report of a thyroid nodule that received US examination should be structured and written in order to avoid colorful descriptive terms. Tappouni et al. suggested an algorithmic approach to stratify thyroid nodules, further aiding radiologists to discriminate benign from suspicious nodules [4].

As documented by Eze et al., FNA-induced reactive changes in thyroid nodules can appear worrisome and may include features such as atypical nuclei, hemorrhage, infarction, fibrinoid necrosis, fibrosis, cystic degeneration, pseudocapsular invasion, and squamous metaplasia that may resemble suspicious imaging findings, resulting in incorrectly classifying a previously aspirated thyroid nodule as TIRADS 4a [22]. Such FNA-induced changes may explain a subset of false-positive TIRADS cases, in which subsequent surgical resection of these surgeries is negative (so-called vanishing tumors).

## 4. Results from Applying TIRADS

Various studies have evaluated the prediction of thyroid malignancy using TIRADS [25,26,27,28,29,30,31,32,33,34,35,36,37,38,39] (Table 3). Shayganfar et al. studied 239 thyroid nodules combining TIRADS and FNA outcome using the Bethesda System for Reporting Thyroid Cytopathology (TBSRTC) [25]. The BSRTC includes six diagnostic categories including: bon-diagnostic (I); benign and bon-beoplastic (II); atypia of undetermined significance or follicular lesion of undetermined significance (AUS/FLUS) (III); follicular neoplasm or suspicious for follicular neoplasm (FN/SFN) (IV); suspicious for malignancy (SM) (V); and malignant (VI) [9]. In their study, the Bethesda system documented that thyroid nodules with TIRADS > 4 and a diameter lower than 12 mm were highly suspicious for malignancy, with a sensitivity of 91.7% and specificity of 52.8%. They found an inverse relationship between nodular size and malignancy risk [25].

Barbosa et al. analyzed the correlation of ACR-TIRADS and ATA guidelines in the evaluation of 140 indeterminate thyroid lesions [26]. According to their study, the combination of US classification, ACR-TIRADS, and ATA along with TBSRTC is useful for detecting benign lesions in Bethesda III nodules and malignant lesions in Bethesda IV/V nodules. The ROM increased according to US suspicion categories (*p* < 0.001) for both US classifications (i.e., TIRADS and ATA). Whilst thyroid nodules with the lowest TIRADS categories had 95.3% sensitivity and 94% negative predictive value (NPV), the highest TIRADS categories were significantly associated with cancer.

Several other studies have also evaluated the use of US patterns to stratify the risk of malignancy for indeterminate thyroid lesions. Grani et al. studied 49 indeterminate lesions combined with TIRADS and ATA systems. They concluded that nodules classified as TIRADS 3 or as having a very low suspicion could be followed-up with FNA, whilst TIRADS 4c nodules had a high positive predictive value (PPV) of 71% with a suggestion for surgical procedure [33]. Moreover, Maia et al. studied 136 indeterminate thyroid lesions combining TIRADS with TBSRTC [28]. They found that Bethesda III nodules with a TIRADS 3 and 4a had high sensitivity (80%) and NPV (90%), implying that conservative management was adequate. On the other hand, thyroid nodules scored as TIRAD 4c and 5 with Bethesda IV and V had a high ROM at 75% and 76.9%, respectively. Rocha et al. investigated 143 indeterminate thyroid lesions, classified as Bethesda III and IV, who were referred to surgery and they hey found a ROM ranging from 0% to 72% [29]. Chaigneau et al. studied 602 indeterminate thyroid nodules classified as TIRADS score 3, 4a, 4b, and 5 with different ROM as 20.5%, 29%, 63.4%, and 100%, respectively [30].

Friedrich-Rust et al. demonstrated promising results in a study including three observers for 114 thyroid nodules [34]. They found that the interobserver agreement was only fair for TIRADS categories 2–5 (Cohen kappa-ck = 0.27, *p* = 0.000001) and TIRADS categories 2/3 versus 4/5 (ck = 0.25, *p* = 0.0020). The NPV was 92–100% for TIRADS categories 4 and 5 in the same study. Valderrabano et al. [31] and Barbosa et al. [26] concluded that there were no differences in the prevalence of malignancy between indeterminate nodules with low or intermediate ATA suspicious patterns, confirming that hypoechogenicity alone did not seem to improve the risk stratification of indeterminate lesions. In contrast, any additional suspicious US feature significantly increases the risk of malignancy of the indeterminate nodules. Rahal et al. assessed a significant association between TIRADS outcome and TBSRTC (*p* < 0.001) in the evaluation of 1000 retrospective thyroid nodules [32]. Benign Bethesda results (95.5%) had been classified as TIRADS 2 or 3, whilst among those classified as TIRADS 4c and 5, the majority belonged to Bethesda VI (68.2% and 91.3%, respectively). Furthermore, among TIRADS 4a–c and 5, the proportion of malignancy was 16%, 43.2%, 72.7%, and 91.3%, respectively. Hence, this study supports the role of TIRADS for the correct assessment and management of thyroid nodules [32]. Zhang et al. studied 319 thyroid nodules combining TIRADS classification and the contrast-enhanced ultrasound (CEUS) enhancement pattern of thyroid nodules concluding that the accuracy in the diagnosis was 96% especially for TIRADS class-4 thyroid nodules [27].

Grani et al. assessed the performance of five internationally endorsed sonographic classification systems [33]. They included 502 cases classified with both the Italian classification system and TBSRTC. The application of the FNA criteria systems reduced the number of biopsies performed by 17.1% to 53.4% for the Italian and TBSRTC, respectively. Among the sonographic risk stratification systems, ACR-TIRADS allowed the largest reduction of biopsies (more than 50%) and the lowest false negative rate (2.2%). Middleton et al., in a multi-institutional reevaluation of thyroid nodules, found that TIRADS was favorably comparable with the ATA and the Korean society of thyroid radiology classifications in reducing the number of biopsies [35].

Other controversial areas for TIRADS include microcarcinoma, growth of nodules, number of nodules to be aspirated and the evaluation of cervical nodes [6]. A paper by Tessler et al. also discussed these issues [6]. Concerning the performance of FNAs for subcentimeter nodules, the ACR-TIRADS agree with other guidelines in limiting FNA of nodules smaller than 1 cm, even if they are highly suspicious. Nevertheless, due to the possibility of active surveillance, ablation, or lobectomy for microcarcinoma, an FNA may be performed.

The committee defined the number of nodules to be biopsied [6]. They recommended one targets no more than two nodules defined by the most worrisome TIRADS. Among the criteria, size should be one of the primary criteria for FNA. Furthermore, the evaluation of cervical lymph nodes is a vital part of every thyroid sonographic examination, and it should be recommended for suspicious nodes.

Another point of discussion is represented by the growth of nodules [6]. The ACR-TIRADS defines a significant enlargement when there is a 20% increase in at least two nodular dimensions and a minimal increase of 2 mm, or a 50% or greater increase in volume, compared with the immediately previous US evaluation [6].

Yang et al. discussed the role of ARC-TIRADS in triaging thyroid follicular cells with papillary-like nuclear features obtained by FNA in order to evaluate the extent of surgery [37]. They found that ACR TI-RADS can be combined with morphology, including NIFTP among cytological diagnoses.

In another paper, Yang et al. studied 179 cases including 72 (40.2%) noninvasive follicular thyroid neoplasm with papillary-like nuclear features (NIFTP), 37 (20.7%) encapsulated FVPTC with invasion (EFVPTC), and 70 (39.1%) infiltrative FVPTC (IFVPTC) without a capsule [38]. They underlined that either NIFTPs or minimally invasive EFVPTC have a circumscribed oval/round border and a hypoechoic rim, and hypervascularity with Doppler. On the other hand, the ultrasound findings for IFVPTCs found at least one of the malignant gray-scale features: markedly hypoechoic, taller-than-wide, microcalcifications, or blurred margins.

Wu studied the same correlation with TIRAD, including 346 thyroid FNAC. They found an overall 0.465 r-value between TI-RADS scores and TBSRTC categories. Furthermore, the comparative analysis between TBSRTC and TIRADS showed that sensitivity, specificity, PPV, NPV, and accuracy are 96%, 53%, 76%, 89%, 79% for TI-RADS vs. 100%, 93%, 96%, 100%, 97% for TBSRTC, respectively (*p* = 0.038) [39].

## 5. Other Thyroid Nodule Ultrasound Scoring Systems

Lee et al. assessed the accuracy of rendering a US diagnosis for benign and malignant solid thyroid nodules using a different classification system comprised of five categories [36] (Table 2). These categories included: malignant, suspicious for malignancy, borderline, probably benign, and benign. The criteria, used for fitting the nodules into the different categories, focused on their hypoechogenicity, nodular margins, microcalcifications, a “taller-than-wider” shape, and associated regional lymphadenopathy. In their series of 103 thyroid lesions, Lee et al. demonstrated that this novel thyroid US system had 86% sensitivity, 95% specificity, 91% positive, and 92% negative predictive values, as well as 92% diagnostic accuracy in discriminating benign from malignant lesions [36]. Nonetheless, the suspicious for malignancy US category had a low diagnostic accuracy value, whilst all malignant US nodules were confirmed to be correctly categorized.

The British Thyroid Association (BTA) in 2014 provided guidelines for US scoring of thyroid nodules (BTA-U score) to assist in the management of thyroid cancer [40]. Briefly, it allows for stratifying thyroid nodules as benign, suspicious, or malignant based on ultrasound appearances termed U1-U5. They include five categories as U1 (normal parenchyma; U2 (benign); U3 (indeterminate); U4 (suspicious); and U5 (malignant). The categories are linked with different management. In fact, U2 nodules do not require FNA or follow-up imaging in the absence of concerning clinical features. The assignation of U3-U5 to nodules require FNA with further management based on resultant cytology, radiology and clinical findings. The US features should be combined with the cytological evaluation and the diagnostic categories. The Royal College of Pathologists in 2009 recommended the subdivision of the Thy-3 (indeterminate) category into Thy-3a (atypia) and Thy-3f (follicular neoplasm) [41,42].

Weller et al. studied 73 consecutive cases evaluated by five sonographers [40]. Their results suggested that there was substantial inter-observer agreement, culminating in 100% sensitivity and negative predictive value, with low specificity (32%) and specificity (34%). On the other hand, a study by Brophy et al. using the BTA system on 151 indeterminate thyroid lesions (Thy3) found no statistically significant differences in the ROM between Thy3a and Thy3f [42].

Ulisse et al. combined the Italian system for classifying thyroid nodules with the TIRADS scoring system in a series of 70 thyroid lesions classified as indeterminate lesions (TIR3A or TIR3B) [43]. The authors reported a different rate of malignancy between TIR3A (13%) and TIR3B (44.5%). They also subclassified their patients into three subgroups showing low (8.3%), indeterminate (21.4%), and high (80%) risk of malignancy. Adoption of the second edition of the Italian cytologic classification system has offered better stratification of malignant risk for indeterminate thyroid lesions [11]. This finding was corroborated by Chng et al. using the BTA system and TIRADS, confirming that the risk of malignancy increased from TIRADS 4A (14.3%), TIRADS 4B (23.1%), TIRADS 4C (87.5%), and TIRADS 5 (100%) [44].

The KSThR published their first recommendation in 2011 for utilizing an US-based diagnosis to assist with the management of thyroid nodules [14]. Subsequently in 2016, a taskforce revised these Korean recommendations [8]. Of note, their changes included revising the US malignancy risk stratification system for thyroid nodules now known as the Korean Thyroid Imaging Reporting and Data System (K-TIRADS), adding a risk stratification system of cervical lymph nodes on the basis of US and computed tomography (CT) features, and recommendations for image-guided ablation of benign thyroid nodules. Their data included a detailed analysis of thyroid nodules encompassing: (1) internal composition (solid, predominantly solid, cystic, predominantly cystic); (2) echogenicity (marked hypoechogenicity, mild hypoechogenicity, isoechogenicity, hyperechogenicity); (3) shape (round to oval, irregular); (4) orientation (parallel, non-parallel); (5) margin (smooth, spiculated, ill-defined); (6) calcification (microcalcification, macrocalcification, rim calcification); (7) halo (present or absent); (8) spongiform (present or not); (9) colloid (present or not); and (10) vascularity (from type 1 to 4). Using this score, the system defines five categories including no nodule, benign category with a <3% ROM, low suspicious category with a 3–15% ROM, intermediate suspicion with a 15–50% ROM, and high suspicion category with >60% ROM [45].

## 6. Conclusions

The adoption of an ultrasound (US) system for classifying thyroid nodules is useful for tailoring the diagnostic approach when evaluating these lesions and combining their workup with FNA biopsy [45,46,47,48]. Accurate categorization of thyroid nodules based on an US classification system, irrespective of whether it is the ACR-TIRADS or an alternative system, may help physicians in predicting their ROM and thus rationalize adequate management. Furthermore, combined analysis including TIRADS in concert with the patient’s age, gender, clinical findings, and thyroid nodule size is essential in determining the pre-FNA ROM. Even if TIRADS or another related US-based system demonstrates satisfactory sensitivity in detecting malignant thyroid nodules, it is unlikely going to replace FNA, as the latter remains the gold standard to define the nature of these nodules, especially when cytomorphology is combined with ancillary molecular testing for indeterminate lesions. However, when TIRADS is combined with US-guided FNA this has been shown to greatly improve the accuracy of diagnosing malignant thyroid nodules.

## Figures and Tables

**Table 1:** Summary of the main features of ultrasound-based thyroid nodule systems.

ACR-TIRADS	Korean System	UK BTA System
TR 1 0 points Benign	K-TIRADS 1: no nodule	U1: No nodule
TR 2 2 points no suspicious	K-TIRADS 2: Benign	U2: Benign hyperechoic or isoechoic with a halo cystic change with ring-down artifact (colloid) microcystic or spongiform appearance peripheral egg-shell calcification peripheral vascularity
TR 3 3 points Mildly suspicious	K-TIRADS 3: Low partially cystic/isohyperechoic with no suspicious features	U3: Indeterminate solid homogenous markedly hyperechoic nodule with halo (follicular lesions) hypoechoic with equivocal echogenic foci or cystic change mixed or central vascularity
TR 4 TR4a = 4 TR4b = 5 TR4c = 6 from 4 to 6 points Moderately suspicious	K-TIRADS 4: Intermediate as for K-TIRADS 3 but with any suspicious features or as for K-TIRADS 5 without suspicious features	U4: Suspicious solid hypoechoic (compared with thyroid) solid very hypoechoic (compared with strap muscles) hypoechoic with disrupted peripheral calcification lobulated outline
TR 5 > 7 points Highly suspicious	K-TIRADS 5: High solid hypoechoic nodule with any suspicious feature	U5 Malignant solid hypoechoic with a lobulated or irregular outline and microcalcification papillary carcinoma solid hypoechoic with a lobulated or irregular outline and globular calcification medullary carcinoma intranodular vascularity taller than wide axially (AP > ML) characteristic associated lymphadenopathy

American College of Radiology Thyroid Imaging, Reporting and Data System (ACR-TIRADS); K-TIRADS: Korean Tirads; UK BTA TIRADS: United Kingdom British Thyroid Association TIRADS. TR = TI-RADS; AP = anteroposterio; ML = mediolateral.

**Table 2:** Criteria adopted for the definition of the TIRADS system score categories.

Criteria	Definitions
Composition	Cystic = 0 Spongiform = 0 Mixed solid and cystic = 1 Solid = 2
Echogenecity	Anechoic = 0 Hyperechoic or isoechoic = 1 Hypoechoic = 2 Very hypoechoic = 3
Shape	Wider-than-tall = 0 Taller-than-wide = 3
Margins	Smooth = 0 Ill-defined = 0 Lobulated or irregular = 2 Extrathyroid extension = 3
Echogenic foci	None or large comet-tail artifacts = 0 Macrocalcifications = 1 Peripheral calcifications = 2 Punctate echogenic foci = 3

**Table 3:** prediction of thyroid malignancy using TIRADS in some of the proposed studies.

Series	N° Cases	Sensitivity	Specificity	PPV	NPV	Diagnostic Accuracy	ROM (Ranged According to the Cytologic Categories)
Shayganfar [25]	239	91.7%	52.8%	/	/	/	0−25%
Barbosa [26]	140	95.3%	84.6%	87%	94%	90.2%	20−92.9%
Zhang [27]	319	86.7%	91.4%	75.6%	95.3%	96%	0−90.5%
Maia [28]	242	80%	84%	71%	90%	66.7%	8.7%−77%
Rocha [29]	143	80.4%	94%	52.4%	95%	/	0−72%
Chaigeau [30]	602	95%%	/	77.6%	55%	/	20−100%
Rahal [32]	1000	/	/	/	/	/	16−92%
Grani [33]	502	83.3%	56.2%	12.8%	97.8%	/	2−20%
Wu [39]	346	96%	53%	76%	89%	79%	

PPV: positive predictive value, NPV = negative predictive value; ROM = risk of malignancy
